# Physical activity shifts gut microbiota structure in aged subjects with overweight/obesity and metabolic syndrome

**DOI:** 10.5114/biolsport.2024.133005

**Published:** 2023-12-20

**Authors:** Patricia Ruiz-Limón, Jananee Muralidharan, Ana M. Gomez-Perez, Mora Murri, Jesús Vioque, Dolores Corella, Montse Fitó, Josep Vidal, Jordi Salas-Salvadó, Laura Torres-Collado, Oscar Coltell, Alessandro Atzeni, Olga Castañer, Mònica Bulló, M. Rosa Bernal-López, Isabel Moreno-Indias, Francisco J. Tinahones

**Affiliations:** 1 Department of Endocrinology and Nutrition, Virgen de la Victoria University Hospital, The Biomedical Research Institute of Malaga and Platform in Nanomedicine (IBIMA BIONAND Platform), University of Malaga, 29016 Malaga, Spain; 2 CIBER in Physiopathology of Obesity and Nutrition (CIBEROBN), Carlos III Health Institute, 28029 Madrid, Spain; 3 University of Rovira i Virgili, Department of Biochemistry and Biotechnology, Human Nutrition Unit, 43003 Reus, Spain; 4 Institute of Health and Biomedical Research of Alicante. University of Miguel Hernández (ISABIAL-UMH), 03010 Alicante, Spain; 5 CIBER Epidemiology and Public Health (CIBERESP), Carlos III Health Institute, 28029 Madrid, Spain; 6 Department of Preventive Medicine, University of Valencia, 46100 Valencia, Spain; 7 Cardiovascular Risk and Nutrition (Regicor Study Group), Hospital del Mar Research Institute (IMIM), 08003 Barcelona, Spain; 8 Endocrinology and Nutrition Department, Clinic Universitary Hospital, 08036 Barcelona, Spain; 9 August Pi i Sunyer Biomedical Research Institute (IDIBAPS), 08036 Barcelona, Spain; 10 Pere i Virgili Health Research Institute (IISPV). San Joan University Hospital, 43003 Reus, Spain; 11 Department of Computer Sciences. University Jaume I, Castellon, Spain; 12 Department of Internal Medicine of Regional University Hospital, Institute of Biomedical Research in Malaga (IBIMA), 29009 Málaga, Spain

**Keywords:** Gut microbiota, Metabolic syndrome, Obesity, Overweight, Physical activity

## Abstract

We aimed to identify how physical activity (PA), within the context of a Mediterranean diet, affects metabolic variables and gut microbiota in older individuals with overweight/obesity and metabolic syndrome. Observational analysis was conducted as part of the PREDIMED-Plus study with 152 males and 145 females with overweight/obesity and metabolic syndrome. General assessments, anthropometric and biochemical measurements, and gut microbial 16S rRNA sequencing data were analyzed at baseline and 1-year of follow-up. Participants were stratified by tertiles of 1-year change in total PA-related energy expenditure ranging from -98.77 to 1099.99 METs (min/week). The total PA percentage of change was reduced in tertile 1 (-44.83 ± 24.94), increased in tertile 2 (28.96 ± 23.33) and tertile 3 (273.64 ± 221.42). Beta diversity analysis showed differences in the gut microbiota population within each tertile group. Significant differences were found at phylum, family, and genus levels in the gut microbiota of the three tertile groups at baseline and 1-year timepoint. Tertile 3, the group with the greatest increase in PA, was characterized by increases in their levels of *Sutterella, Bilophila*, and *Lachnospira* bacteria as well as a reduction in *Collinsella*. Moreover, this tertile showed a different pattern in its predicted metabolic capacities to the other groups. Our results have demonstrated that changes in PA such as lifestyle and Mediterranean diet induces specific variations in the gut microbiota profile. This modulation of gut microbiome populations and their metabolic capacities may contribute to the health of the aged individuals with overweight/obesity and metabolic syndrome.

## INTRODUCTION

Regular physical activity (PA) has significant health benefits, like reduction of cardiovascular risk, and improving cardiometabolic risk factors, such as reducing body mass index (BMI) and fat mass, fasting glucose, and insulin levels. Several studies have indicated that PA attenuates the risk of obesity and might be a strategy to treat obesity with numerous cardiovascular benefits [1]. However, a great increase in sedentary behaviors and physical inactivity have irrupted in the last decades, particularly in older adults [2], which is associated with weight gain and an increased incidence of obesity and obesity-related comorbidities [3]. For that reason, lifestyle interventions are of paramount importance at those ages for healthy aging. In previous work, in the frame of PREvención con DIeta MEDiterránea (PREDIMED)-Plus, a trial designed to evaluate the long-term effectiveness of an intensive weight loss lifestyle intervention on primary cardiovascular prevention [4], demonstrated that the intervention resulted in an effective increase in daily PA in older adults.

Recent findings pointed out that PA can determine changes in the gut microbiota composition, playing a positive role in energy regulation [5]. PA can enrich microbial biodiversity and enhance the number of beneficial microbial species [5], like the phylum *Verrucomicrobia*, which includes bacteria related to better body composition and improved metabolic health [6]. Also, PA could increase the synthesis of metabolites involved in the good maintenance of intestinal health. Additionally, several studies on subjects with overweight/obesity have shown a slight increase in the abundance of *Actinobacteria, Firmicutes, Proteobacteria*, and *Verrucomicrobia* phyla and *Bacteroides* family after PA [7]. Moreover, another study suggested that an adequate PA could modulate the gut microbiota in elderly individuals [8], maintaining health, although different PA behaviors have shown diverging effects on both, the beta diversity, and the relative abundance of specific bacteria in the gut [9]. Although a growing body of evidence is pointing out the beneficial effect of PA in the modulation of gut microbiota, this intriguing pattern is still not fully elucidated, especially in older adults, when body physiology changes. We hypothesized that the increase of PA as a part of a lifestyle intervention results in changes in gut microbiota composition related to improvements in metabolic parameters. Thus, the objective of the current study is to identify how an increase in the total PA, within a context of a Mediterranean lifestyle, affects metabolic parameters and gut microbiota in older individuals with overweight/obesity and metabolic syndrome.

## MATERIALS AND METHODS

### Study design and participants

This sub-study in the context of the PREDIMED-Plus trial, a multicenter, randomized trial, to assess the effect of a weight-loss intervention program based on an energy-restricted traditional Mediterranean diet, PA promotion, and behavioral support, in comparison with a usual care intervention only with the energy-unrestricted Mediterranean diet. This study was registered at the International Standard Randomized Controlled Trial (ISRCT; http://www.isrctn.com/ISRCTN89898870) with the number 89898870 and the date of 24 July 2014. More details of the PREDIMED-Plus study protocol are fully described and available at http://predimedplus.com [10]. This sub-study was a longitudinal analysis focused on the baseline and 1-year timepoints.

PREDIMED-Plus eligible participants were men and women (aged 55–75 years), with overweight/obesity (body mass index (BMI) ≥ 27 and ≤ 40 kg/m^2^) and metabolic syndrome (MetS). Briefly, exclusion criteria included a previous history of cardiovascular disease, any chronic medical condition, acute infectious processes, psychiatric disorders, alcohol and drug abuse, institutionalization, use of specific medications, relevant recent weight loss, any food allergy to Mediterranean diet food and the use of antibiotic therapy, probiotic or prebiotic in the previous three months. All participants provided written informed consent, and the study protocol and procedures were approved according to the ethical standards of the Declaration of Helsinki by all the participating institutions.

The present sub-study included 152 men and 145 women, from two PREDIMED-Plus recruiting centers (Malaga and Reus (Spain)) with stool samples available at baseline and after 1-year of intervention as well as available PA information. The type of activity, frequency, and duration (minutes/day) of PA were self-reported using the validated REGICOR Short Physical Activity Questionnaire [11]. PA was defined as the sum of total minutes and hours daily of 6 types of activities performed during a month (brisk walking, walking at a slow/ normal place, walking in the countryside, climbing stairs, working in the garden, exercising, or playing sports at home, outdoors or in a gym). Then, total PA was determined as the sum of the total activities according to the Compendium of Physical Activities [12]. Total PA-related energy expenditure was calculated as the summed product of frequency, duration, and intensity of each activity multiplied by 7, obtaining the metabolic equivalent of tasks (METs) min/week [13].

The observational study was performed by stratifying the participants by tertiles of change in PA-related energy expenditure after 1-year intervention, with the following tertile groups T1 (n = 99), T2 (n = 99), and T3 (n = 99). The changes in the variable total PA-related energy expenditure were expressed as percentages and were calculated between basal and the 1-year differences, and divided by the basal.

### Anthropometric, Laboratory Variables, and Samples Collection

At baseline, and 1-year follow-up visits, waist circumference, weight, and height were measured, and systolic blood pressure (SBP) and diastolic blood pressure (DBP) were measured using a validated semiautomatic oscillometer (Omron HEM-705CP, Kyoto, Japan). Peripheral venous blood and fecal samples were collected, at both time points, after overnight fasting. Serum glucose, total cholesterol, high-density lipoprotein (HDL) cholesterol, and triglycerides were measured by standard enzymatic methods. Low-density lipoprotein (LDL) cholesterol was calculated by the Friedewald formula. Glycated hemoglobin (HbA1c) was measured by a chromatographic method.

### Fecal DNA Extraction and 16S Sequencing

DNA extraction from stools was performed using the QIAamp DNA stool Mini kit (Qiagen, Hilden, Germany) following the manufacturer’s instructions. DNA concentration and purity were determined using a Nanodrop spectrophotometer (Nanodrop Technologies, Wilmington, DE, USA).

The Ion 16S Metagenomics Kit and Ion Plus Fragment Library Kit (Thermo Fisher Scientific Inc., Waltham, MA, USA) were used to build the sequencing libraries from the 16S rRNA gene. Emulsion PCR and sequencing of the amplicon libraries were performed using the Ion Chef System and Torrent S5^TM^ system, respectively (Thermo Fisher Scientific) according to the manufacturer’s instructions.

### Bioinformatic Analysis

Torrent Suite™ Server software (Thermo Fisher Scientific), version 5.4.0, with default parameters for the 16S Target Sequencing (bead loading ≤ 30, key signal ≤ 30, and usable sequences ≤ 30) was used to base calling and run demultiplexing. Quality sequences were further translated into amplicon sequence variants (ASVs) using DADA2 with adapted parameters for Ion Torrent data within open-source Quantitative Insights into Microbial Ecology (QIIME2, version 2019.10) [14], which were also used for diversity analysis with the diversity plugin. Alpha diversity indexes (observed features, pielou-evenness, and faith’s PD) were calculated within each group and between the groups by Kruskal-Wallis test. Weighted and Unweighted Unifrac distance matrices were calculated and permutational multivariate analysis of variance (PERMANOVA) was used looking to look for differences in group compositions. Taxonomic assignment was performed through clustering with VSEARCH and the reference base Greengenes version 13_8 at 97% of identity. To perform the differential abundance analysis within each tertile between the baseline and 1-year timepoints, ASV counts and taxonomic information generated with QIIME2 were imported into the MicrobiomeAnalyst webtool [15], where the data filtering and normalization steps were performed. Differential abundance analyses were assessed with MetagenomeSeq within MicrobiomeAnalyst with the default parameters of the developer. On the other hand, the longitudinal analysis, to perform the differential abundance analysis between microbiota changes among tertiles, was assessed through a volatility analysis, by using the q2-longitudinal plugin to search for important features, and analyzed to establish statistical significance within the same plugin. Phylogenetic Investigation of Communities by Reconstruction of Unobserved States plugin (PICRUSt2) was used to predict metagenome function within QIIME2. MetaCyc pathways were normalized within QIIME2 and further analyzed with STAMP with Welch’s t-test option.

### Statistical Analysis

Statistical analysis was performed with IBM SPSS Statistics 25 (IBM, Armonk, NY, USA). Quantitative variables were expressed as mean ± standard deviation (SD) for normally distributed data and as median ± interquartile range (IQR) for non-normally distributed data and percentages for categorical variables. The population was stratified by tertiles of change in total physical activity-related energy expenditure like lifestyle after the 1-year intervention. The normal distribution of variables was assessed using the Kolmogorov–Smirnov test. The bivariate analysis was performed using paired Student’s tests for continuous data or the Wilcoxon test for non-normally distributed data. Differences across groups were evaluated through one-way analysis of variance (ANOVA) for continuous data or Krus-kal–Wallis’s test for non-normally distributed data. Categorical data were analyzed using Pearson’s chi-square test. Correlations were assessed by Spearman´s rank correlation. Values were statistically significant when *p* < 0.05. Regarding the microbiota analysis, the statistically significant threshold was established at *p* ≤ 0.05 and FDR-corrected *q* < 0.2 to formulate the possible hypothesis.

## RESULTS

### Characteristics of the study population

Table 1 shows the information relative to the anthropometric and laboratory and blood pressure results of the study. In general, after the 1-year intervention, a significant decrease in weight and waist circumference was observed in all three groups, as well as, significant differences were observed in the glucose, triglycerides, HDL, and HbA1c in the T3 group after 1-year of intervention. We observed lower levels of PA in the T3 group *versus* T1 and T2 groups at baseline, as well as a greater percentage of total PA change after 1-year of intervention in T3 (*p* < 0.001). Although participants belonged to a Mediterranean country, the baseline Mediterranean adherence score was equal to or below the median Mediterranean adherence score [low (≤ 7), medium (8-10), and high (11-17)] in the three groups. This adherence augmented after 1-year of intervention, with significantly greater improvements in the T3 group *versus* T2 and T1 groups (*p* = 0.005). Concerning changes at 1-year in anthropometric variables, participants from the three groups decreased an average of -2.85 ± 4.24 kg of body weight, with a greater weight loss in T3 participants (*p* = 0.005). BMI, waist circumference, and hip circumference changes decreased in the three groups, but significant differences in changes were observed between T2 and T3 compared to T1 in hip circumference (*p* = 0.002). T3 decreased significantly their waist circumference and BMI to T1 participants (*p* < 0.001 and *p* = 0.020, respectively). Finally, T3 participants decreased their LDL-cholesterol *versus* T2 and T1 participants, although translated into changes showed only a trend towards statistical significance (*p* = 0.051).

**TABLE 1:** Clinical and laboratory characteristics at baseline and 1-year of intervention according to study total physical activity tertile groups.

		T1 (n = 99)	T2 (n = 99)	T3 (n = 99)	*p-value* across tertiles ^&^
Variables					

Age (years)		65.00 ± 5.02	64.45 ± 4.64	64.42 ± 5.50	0.740

Sex (male/female)		45/54	51/48	56/43	0.293

Weight (Kg), mean ± SD	Baseline	85.77 ± 12.21	88.55 ± 12.06	89.69 ± 12.67 ^a^	0.072
	1-year	84.03 ± 12.41^*^	86.20 ± 11.87 ^*^	86.14 ± 12.87 ^*^	0.435
	Change	-2.04 ± 3.75	-2.57 ± 4.48	-3.95 ± 4.51 ª^, b^	0.005

Waist circumference (cm), mean ± SD	Baseline	107.70 ± 9.72	109.86 ± 9.34	110.12 ± 9.12 ^a^	0.140
	1-year	106.25 ± 10.48 ^*^	107.30 ± 9.78 ^*^	106.11 ± 10.16 ^*^	0.688
	Change	-1.34 ± 4.19	-2.27 ± 4.84	-3.60 ± 5.30 ^a^	< 0.001

Hip circumference (cm), median (IQR)	Baseline	109.0 (103.9–115.3)	109.0 (103.5–114.5)	110.5 (104.0–116.0)	0.711
	1-year	108.0 (102.5–114.3) ^*^	108.9 (103.0–114.5)	107.0 (102.0–113.0) ^*^	0.333
	Change	-0.72 ± 4.01	-0.20 ± 4.53	-2.40 ± 4.19 ^a, b^	0.002

Waist-Hip ratio, mean ± SD	Baseline	0.98 ± 0.07	1.00 ± 0.07	0.99 ± 0.07	0.159
	1-year	0.98 ± 0.07	0.98 ± 0.07 ^*^	0.98 ± 0.07 ^*^	0.628
	Change	-0.53 ± 4.39	-1.99 ± 4.49	-1.17 ± 4.82	0.055

BMI (Kg/m^2^), median (IQR)	Baseline	32.3 (29.6–35.1)	32.8 (30.2–35.4)	32.7 (31.1–35.9)	0.205
	1-year	31.5 (29.5–34.7) ^*^	32.1 (29.4–34.5)	31.7 (29.5–35.0) ^*^	0.883
	Change	-1.99 ± 3.48	-2.50 ± 4.64	-3.68 ± 4.42 ^a^	0.020

Glucose (mg/dL), median (IQR)	Baseline	103.0 (93.0–116.0)	101.0 (91.0–118.0)	104.0 (95.0–117.0)	0.561
	1-year	101.0 (94.0–114.0)	101.0 (91.0–119.0)	101.0 (91.0–113.0) ^*^	0.841
	Change	-0.94 ± 13.35	0.83 ± 16.62	-2.06 ± 17.95	0.192

Triglycerides (mg/dL), median (IQR)	Baseline	162.0 (117.0–232.0)	151.0 (103.0–196.0)	152.0 (118.0–210.0)	0.345
	1-year	153.0 (109.0–203.0)	135.0 (103.7–177.5)	137.0 (109.0–184.0) ^*^	0.323
	Change	2.34 ± 44.74	-2.20 ± 32.98	0.76 ± 68.39	0.441

Total cholesterol (mg/dL), median (IQR)	Baseline	197.0 (174.0–225.0)	199.0 (172.0–223.0)	197.0 (178.0–223.0)	0.760
	1-year	195.0 (174.0–223.0)	202.0 (167.7–228.0)	192.0 (175.0–219.0)	0.853
	Change	1.02 ± 17.92	0.66 ± 15.83	-0.98 ± 14.62	0.354

HDL (mg/dL), median (IQR)	Baseline	47.0 (40.0–52.0)	48.0 (42.0–57.0)	46.0 (41.0–55.0)	0.372
	1-year	49.0 (41.0–58.0) ^*^	49.0 (42.0–59.0)	51.0 (44.0–58.0) ^*^	0.601
	Change	5.68 ± 12.00	2.48 ± 18.97	8.69 ± 17.71	0.093

LDL (mg/dL), median (IQR)	Baseline	111.0 (97.0–130.0)	114.0 (91.7–140.2)	117.0 (102.0–138.0)	0.333
	1-year	113.0 (91.0–135.0)	118.5 (96.5–145.2)	113.0 (99.0–131.0)	0.488
	Change	3.23 ± 30.38	4.05 ± 21.45	-1.40 ± 22.86	0.051

HbA1c (%)	Baseline	6.07 ± 1.10	6.10 ± 0.85	6.02 ± 0.68	0.206
	1-year	5.95 ± 0.89	6.07 ± 1.11	5.91 ± 0.64 ^*^	0.298
	Change	-2.23 ± 13.93	-1.40 ± 14.27	-1.38 ± 6.73	0.496

SBP (mm Hg), median (IQR)	Baseline	139.0 (130.0–149.7)	138.3 (128.0–146.3)	138.3 (128.3–149.3)	0.526
	1-year	136.3 (124.3–146.3) ^*^	136.7 (125.7–146.7)	133.7 (125.3–146.0)	0.961
	Change	-2.98 ± 9.94	-1.27 ± 11.18	-1.90 ± 10.85	0.523

DBP (mm Hg), mean ± SD	Baseline	79.00 ± 9.94	80.11 ± 8.60	80.17 ± 10.78	0.642
	1-year	76.72 ± 9.18 ^*^	78.13 ± 8.14 ^*^	78.34 ± 10.72 ^*^	0.440
	Change	-2.29 ± 10.24	-1.79 ± 11.13	-1.82 ± 9.90	0.930

17-point Mediterranean adherence score, median (IQR)	Baseline	8.0 (6.0–9.0)	8.0 (6.0–10.0)	8.0 (6.0–10.0)	0.785
	1-year	12.0 (10.0–14.0) ^*^	13.0 (10.0–15.0) ^*^	14.0 (11.0–16.0) ^*, a^	0.002
	Change	3.93 ± 3.03	4.14 ± 3.63	5.27 ± 3.48 ^a^	0.005

Total physical activity (METs-min/week), median (IQR)	Baseline	3338.0 (1766.9–5048.9)	2125.8 (1165.5–3356.6) ^a^	1021.9 (524.4–1874.1) ^a, b^	< 0.001
	1-year	1524.4 (671.3–2564.1) ^*^	2937.0 (1342.6–4510.5) ^*, a^	3416.3 (1958.0–5272.7) ^*, a^	< 0.001
	Change	-44.83 ± 24.94	28.96 ± 23.33 ^a^	273.64 ± 221.42 ª^, b^	< 0.001

BMI, body mass index; DBP, diastolic blood pressure; HbA1c, glycated hemoglobin; HDL, high-density lipoprotein; IQR, interquartile range; LDL, low-density lipoprotein; METs, metabolic equivalent of tasks; SBP, systolic blood pressure; SD, standard deviation. Participants were divided by tertiles of 1-year change in total PA-related energy expenditure, between -98.77 and -10.24 for T1, between -10.25 and 69.10 for T2, and between 69.11 and 1099.99 for T3.

* p ≤ 0.05 baseline vs. 1-year of intervention value, according to paired Student’s tests or Wilcoxon tests.

& One-way ANOVA, Pearson’s chi-square test or Kruskal–Wallis test used to calculate differences across tertiles; Pearson’s chi-square test, Student’s t-test or Mann–Whitney test used to calculate differences between tertiles;

a p ≤ 0.05 vs. T1;

b p ≤ 0.05 vs. T2.

### Changes in gut microbiota within each group after 1-year of intervention

**FIG. 1:**
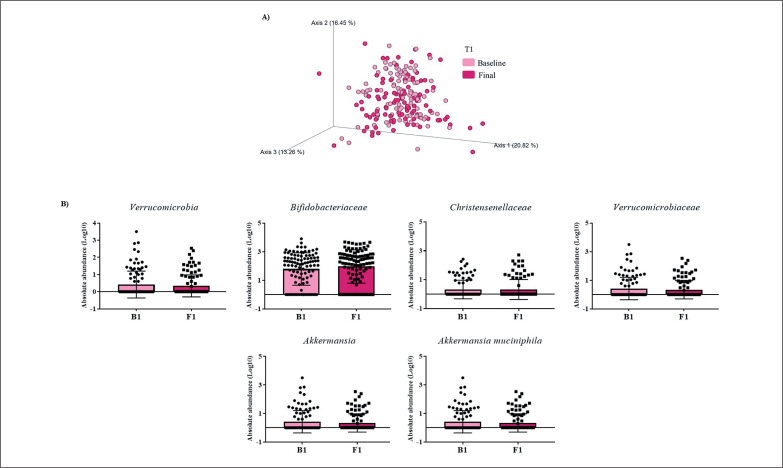
Changes in gut microbiota within the T1 group. A) Significant changes in PCoA of weighted UniFrac distances (Baseline: grey diamonds; final point: black dots). B) Significant changes in the abundance of the interest bacteria evaluated with MetagenomeSeq and having a *p* < 0.05 and *q* < 0.2, at phylum, family, genus, and species levels.

**FIG. 2:**
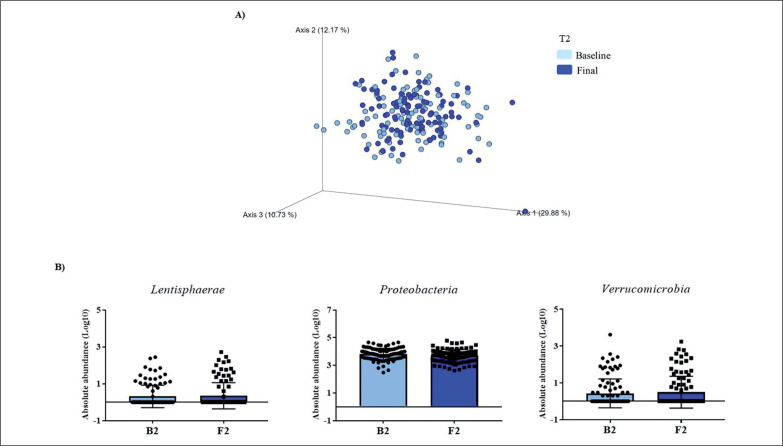
Changes in gut microbiota within the T2 group. Clustering of fecal bacterial communities according to the different study groups by PCoA using weighted UniFrac distances. A) Significant changes in PCoA of weighted UniFrac distances (Baseline: grey dots; final point: black rings). B) Significant changes in the abundance of the interest bacteria evaluated with MetagenomeSeq and with a *p* < 0.05 and *q* < 0.2, at the phylum level.

**FIG. 3:**
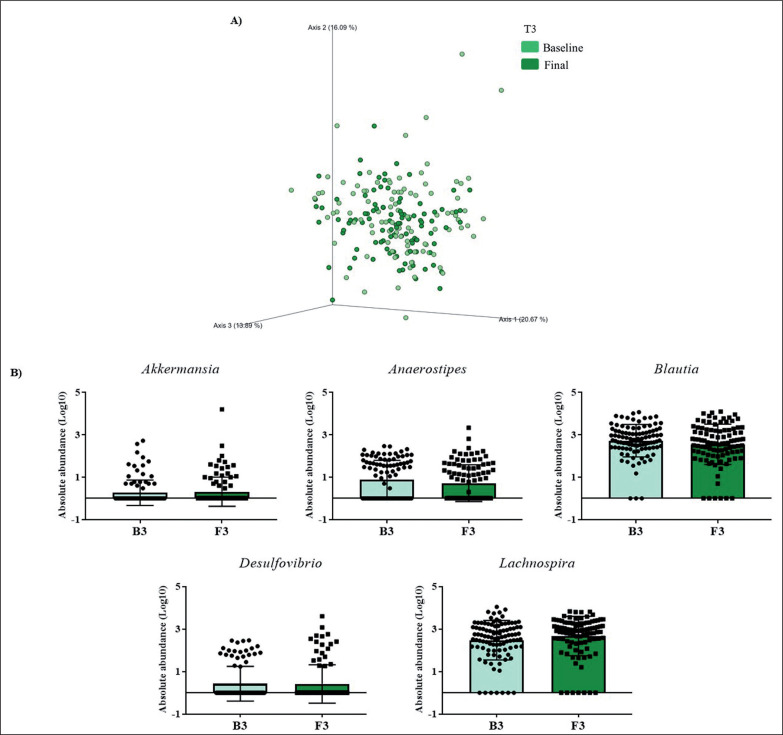
Changes in gut microbiota within the T3 group. Clustering of fecal bacterial communities according to the different study groups by PCoA using weighted UniFrac distances. A) Significant changes in PCoA of weighted UniFrac distances (Baseline: grey cones; final point: black squares). B) Significant changes in the abundance of the interest bacteria evaluated with MetagenomeSeq and having a *p* < 0.05 and *q* < 0.2, at the genus level.

Gut microbiota populations changed within each group according to weighted UniFrac distances: T1 group (*p* = 0.042; Figure 1A), T2 group (*p* = 0.009; Figure 2A), and T3 group (*p* = 0.046; Figure 3A), although this trend was not observed in the unweighted version. On the other hand, no differences were observed according to alpha diversity assessments, using phylogenetic diversity (Faith_pd), richness community (Observed features), and homogeneity (Pielou-Evenness) (Supplementary Table 1).

The MetagenomeSeq analysis was used to evaluate the gut microbiota features changes comparing baseline and 1-year timepoint of the T1, T2, and T3 groups. The T1 group of participants was characterized by a decrease in the level of *Verrucomicrobia* (*p* < 0.001; *q* = 0.004), *Verrucomicrobiaceae* (*p* < 0.001; *q* = 0.018), *Akkermansia* (*p* < 0.001; *q* = 0.019), as well as its species *A. muciniphila* (*p* = 0.003; *q* = 0.059). While, *Christensenellaceae* (*p* = 0.003; *q* = 0.044) and *Bifidobacteriaceae* (*p* = 0.020; *q* = 0.173) were significantly expanded at 1-year time point (Figure 1B). Participants in the T2 group increased their levels of *Lentisphaerae* (*p* < 0.001; *q* = 0.001) and decreased *Verrucomicrobia* (*p* = 0.0017; *q* = 0.005) and *Proteobacteria* (*p* = 0.035; *q* = 0.071) (Figure 2B). Finally, the T3 participants increased their levels of *Lachnospira* (*p* = 0.042; *q* = 0.203), *Desulfovibrio* (*p* = 0.008; *q* = 0.129) and *Akkermansia* (*p* = 0.025; *q* = 0.174) at 1-year timepoint. On the other hand, the T3 group decreased their levels of *Anaerostipes* (*p* < 0.001; *q* = 0.003), *Blautia* (*p* = 0.022; *q* = 0.174), and *Collinsella* (*p* = 0.030; *q* = 0.174) through the time (Figure 3B).

### Changes in gut microbiota between groups

**FIG. 4:**
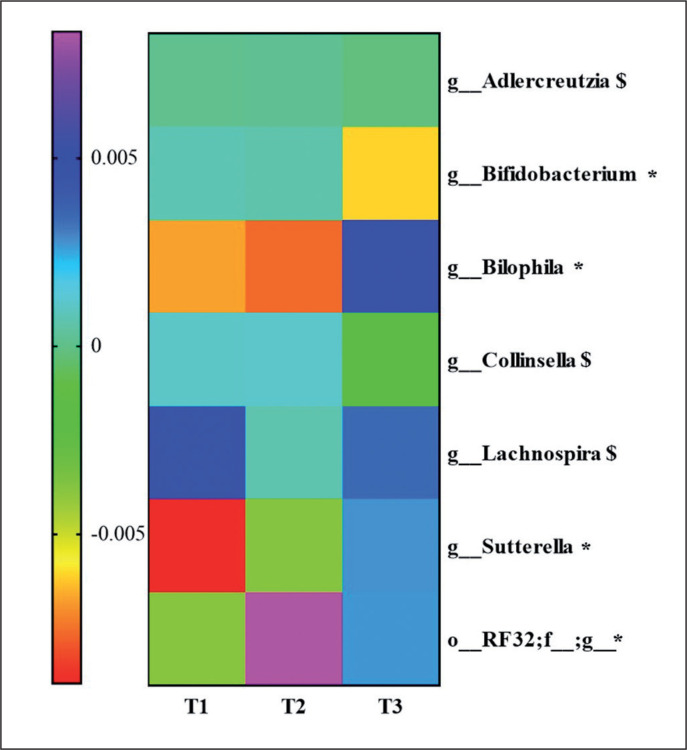
Heatmap for genera that have shown statistical differences between the three physical activity tertile groups. Significantly differences *p < 0.05 and, $ *p* < 0.1.

To know the most relevant taxa according to the changes observed during the lifestyle intervention, a volatility analysis was performed. This volatility analysis, with an accuracy of the model at the genus level of *p* < 0.1, showed 30 important genera. Thus, these genera were subjected to a paired analysis between the three groups of study resulting in four genera significantly different (*p* < 0.05) and three genera tended to differ (*p* < 0.1) between the groups. *Bifidobacterium* abundance decreased in T3 group compared to T2 (*p* = 0.04; *q* = 0.06) and T1 (*p* = 0.02; *q* = 0.06). *Collinsella* and *Adlercreutzia* abundances decreased in the T3 group compared to T2 (*p* = 0.03; *q* = 0.1 and *p* = 0.03; *q* = 0.09, respectively). *Lachnospira* abundance increased in the T3 group compared to the T2 group (*p* = 0.02; *q* = 0.08). An unclassified genus of order *RF32* abundance increased in the T3 compared to T1 (*p* = 0.04; *q* = 0.07) and increased in the T2 group compared to T1 (*p* = 0.007; *q* = 0.02). *Sutterella* abundance was higher in the T3 compared to T2 (p = 0.06; q = 0.09) and T1 (*p* = 0.01; *q* = 0.04), and *Bilophila* abundance was higher in the T3 group compared to the T2 group (*p* = 0.009; *q* = 0.01) and T1 group (*p* = 0.01; *q* = 0.01). These results are summarized in Figure 4.

### Correlation studies between changes in gut microbiota and clinical parameters

**FIG. 5:**
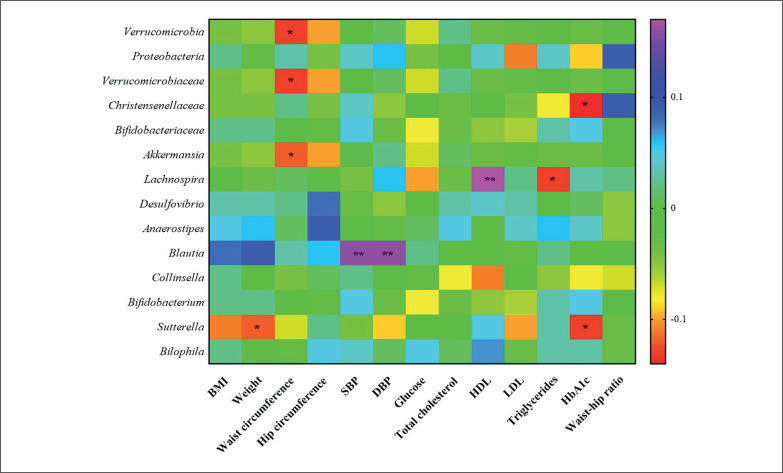
Heatmap representing correlations in the total study population (n = 297) between changes in gut microbiota and clinical variables. Significantly differences *p < 0.05 and **p < 0.01.

Correlation studies demonstrated that the changes in *Verrucomicrobia*, its family *Verrucomicrobiaceae* and its genus *Akkermansia,* were negatively associated with changes in waist circumference. *Christensenellaceae* and the genus *Sutterella* were negatively associated with changes in HbA1c. *Sutterella* was negatively associated with weight changes, as well. The genus *Lachnospira* was positively associated with changes in HDL and negatively with changes in LDL and the genus *Blautia* was positively associated with changes in SBP and DBP (Figure 5).

### Differences in the Metabolic Profiles of Gut Microbiota

**FIG. 6:**
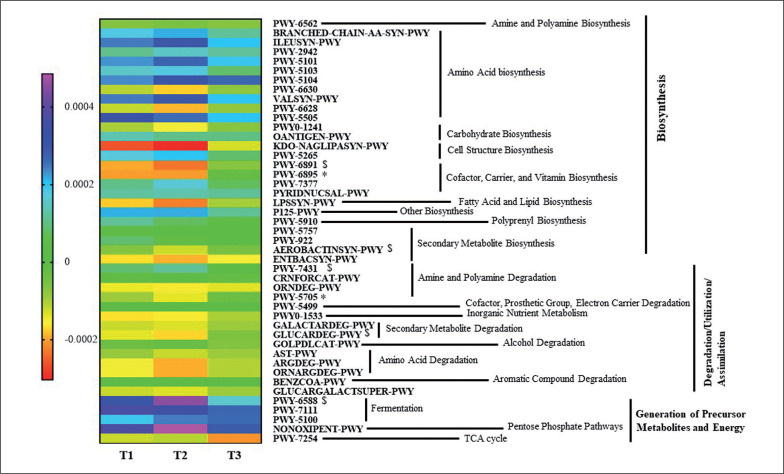
Heatmap representing median values of significantly increasing or decreasing predicted metagenome pathways between the three groups. *Indicates significant differences between groups (*p* < 0.05) and, $ indicates *p* < 0.1 in multiple group tests with the Kruskal Wallis test.

An ANOVA analysis showed that 42 pathways differed between groups in some way (*p* < 0.05, *q* < 0.2). These pathways mainly belonged to roles relative to Biosynthesis, Generation of Precursor Metabolites and Energy, and Degradation/Utilization/Assimilation. These pathways were further analyzed to study their changes between groups with a pairwise analysis (Figure 6, Supplementary Table 2). Although the heatmap clearly shows a different pattern between group T3 and T1 and T2 groups, only some pathways reached at least a statistical tendency when changes were compared. According to the Kruskal Wallis test, allantoin degradation to glyoxylate III (PWY-5705) and the superpathway of thiamine diphosphate biosynthesis II (PWY-6895) showed statistically different changes between groups (*p* < 0.05), whilst *D*-glucarate degradation I (GLU-CARDEG-PWY), aromatic biogenic amine degradation (PWY-7431), pyruvate fermentation to acetone (PWY-6588), thiazole component of thiamine diphosphate biosynthesis II (PWY-6891), and aerobactin biosynthesis (AEROBACTINSYN-PWY) showed a tendency to differ between groups (*p* < 0.1).

## DISCUSSION

In the current study, we observed metabolic benefits associated with an increase in PA in an older adult population. Moreover, changes in PA were associated with specific variations in the gut microbiota profile and its potential functionality accordingly, which could have influenced the improvement of the metabolic variables of the patients.

Recent research has demonstrated that exercise-related physiological remodeling might be extended to gut microbiota [16]. Through weighted UniFrac distances, our study has shown that the gut microbiota population changed differently with PA over a period of 1-year. Our results were in line with works in which α-diversity remained unchanged but the overall community composition (β-diversity) changed after PA intervention [17]. Additionally, others have reported that exercise might increase α-diversity [18], but despite these controversial results, PA has been proposed as a therapeutic approach for obesity and/or hypertension through the modulation of gut microbiota [19].

The differences found in the gut microbiota population indicated different shifts in the abundance of features more than a different profile. Indeed, the T1 group was characterized by an increase of *Bifidobacteriaceae* and *Christensenellaceae* families, which have been associated with leanness, and have been proposed as a marker of healthy aging and longevity [20], which could be the result of adherence to the Mediterranean lifestyle. The levels of the *Verrucomicrobia* phylum, as well as its family *Verrucomicrobiaceae* and its genus *Akkermansia*, were reduced in the T1 group, in the same manner as *Verrucomicrobia* phylum in the T2 group. A lower proportion of *Verrucomicrobia* has been reported in subjects with obesity [21]. Nevertheless, the T3 group presented an increased level of *Akkermansia* after 1-year of intervention. Thus, PA could increase the levels of *Akkermansia* in accordance with other reports [22]. Moreover, we have observed that these changes in *Verrucomicrobia*-related features could be directly implicated in the metabolic improvement of the study subjects because were negatively associated with changes in waist circumference.

The T2 group displayed a reduction in levels of *Proteobacteria*. A raise in *Proteobacteria* has been reported in individuals with obesity and was related to inflammation [23]. Thus, even a small increase of PA, as it occurs in T2, could be related to a betterment in the inflammatory milieu. Additionally, we have detected an increased level of *Lentisphaerae* phylum in this group, but little is known about this minority phylum, although it has been related to *Verrucomicrobia* [24] in terms of health benefits.

The group with the highest increase in PA (T3) showed the greatest changes in its microbiota population. We detected an increase in the level of the genera *Lachnospira* and *Desulfovibrio* at 1-year of intervention. *Desulfovibrio* belongs to the sulfate-reducing bacteria (SRB), which produces hydrogen sulfide (H_2_S), a key intestinal metabolite with diverse effects on host health. Thus, H_2_S released by bacteria in the colon may also contribute to the control of arterial blood pressure [25] and, although the T3 group has not shown statistically significant differences in blood pressure, we observed a slight decrease after 1-year of PA. An increase in the abundance of *Bilophila* was also observed in the T3 group, a genus observed enriched in high-performing individuals [26]. Additionally, like the current study, a recent PREDIMED-Plus sub-study has shown that an increased abundance in *Lachnospira* and *Lachnospiraceae NK4A136* group were noticed in a frame of a Mediterranean lifestyle [27].

On the other hand, the genera *Collinsella* and *Blautia* decreased their levels in the T3 groups. In line with our study, Cancello R. *et al.* observed a decline in *Collinsella* levels after 15 days of the rehabilitation program and a short-term dietary intervention in elderly women with obesity [28]. Moreover, elevated levels of *Collinsella* have been reported to be associated with high levels of total and LDL cholesterol [29]. In agreement with our findings, the T3 group displayed lower levels of LDL and total cholesterol, although these changes were not significant. For its part, *Blautia* is a controversial bacterium in the context of obesity, as *Blautia* has been associated both directly and inversely with obesity [30]. Contrary to our results, others have reported an increase in *Blautia* levels with exercise training although in children [31]. These contradictory results about *Blautia* could be due to the great diversity among *Blautia* oligotypes, indicating that this genus comprises strains with a variety of metabolic capacities developed to a host and a host environment [32].

A decrease in the abundance of *Bifidobacterium* was detected in the T3 group. The intake of *Bifidobacterium* probiotics by athletes has been related to improving exercise physiological adaptation [33], so *Bifidobacterium* could be compromised in some manner with an increase in PA. In addition, an increased abundance of the *Sutterella* genus was observed in the T3 group, in accordance with another study that demonstrated that an unclassified taxon from the *Sutterella* genus had a key role in the transition from sedentary to active individuals [34]. Moreover, we observed a negative association between changes in the abundance of *Sutterella* with changes in weight and HbA1c. Studies have demonstrated that the abundance of *Sutterella* was negatively associated with obesity [35]. However, other findings pointed out *Sutterella* was positively associated with metabolic syndrome [36]. More in-depth studies are needed to understand the contradictory findings of *Blautia, Bifidobacterium,* and *Sutterella*.

Our functionality prediction analysis pointed out that the group which the greatest increase in PA (T3) showed an increase in vitamin metabolism and cofactors such as thiamine (vitamin B1). Some findings have shown that PA may increase the body’s need for thiamine [37], and in this way, there are vitamin B1-producing bacteria with the complete vitamin B1 synthesis pathway, which includes pathways for the synthesis of thiazole and pyrimidine [38]. Indeed, the T3 group involves a higher degradation of various substrates as sources of energy and nutrients of sugars, including increased degradation of sugars such as D-glucarate and amines such as allantoin, which is supported by Yu D. *et al.* who observed in their healthy diet group the same metabolic changes [39]. Additionally, the T3 group significantly decreased its abundance of branched-chain amino acids (BCAAs) after the PA protocol. BCAAs are involved in muscle growth, muscle soreness, and exercise fatigue reductions, interesting features for those who increase their PA. These changes in metabolism according to the level of PA could agree with a study in which patients with prediabetes reported significant differences in the gut microbiome composition after a 12-week aerobic exercise intervention [40]. Moreover, the T3 group significantly decreased their abundance of short-chain fatty acids (SCFAs), and although the decrease of SCFAs could reveal contradictory findings, a study of Muralidharan *et al*. is in line with our findings, and they observed a decrease in predicted pathways related to SCFAs with a lifestyle intervention directed to weight loss [27]. These results further support that PA might mediate effects on human metabolism through changes in the gut microbiome.

The current study has multiple strengths like the fact of being built under a great populational study like PREDIMED-Plus with complete phenotyping of the volunteers. However, it is also subject to a series of limitations. First, the multifaceted intervention strategy whose outcome inference cannot be fully associated with a single component of the intervention. Second, the activity was calculated from a questionnaire self-administered, which could overestimate, or underestimate the values. Nevertheless, the use of a REGICOR questionnaire is a reliable and validated method that records information about leisure and occupational time and the four dimensions of PA including type of activity, frequency, duration, and intensity of PA, as well as sedentary behavior [11]. Third, body composition was assessed with waist circumference, weight, and height, having obtaining more information with other techniques, such as impedanciometry or Dual X-ray Absorptiometry. Fourth, due to the sub-study characteristic of the study, results are focused on changes in the variables during a well-followed-up intervention cohort to avoid baseline differences. Fifth, the aging of the participants needs from other similar studies to extrapolate these results to the whole population, as well as from additional omics analysis that permit more complete information. Lastly, 16S sequencing limits the taxonomy profiling to genus-level resolution, and consequently, it is not possible to determine the pathways associated with the observed relationships. Notwithstanding, 16S sequencing offers the advantage of analyzing a substantial number of samples, benefiting from established and well-curated databases. However, these results point out an implication of the gut microbiome in the improvements associated with an increase in PA that deserves to be further investigated with cohorts specially chosen for this hypothesis.

## CONCLUSIONS

Taken together our results have demonstrated that changes in PA such as lifestyle and Mediterranean diet induces specific variations in the gut microbiota profile. Moreover, this study identifies that the modulation suffered by the gut microbiome populations and their metabolic capacities may contribute to the health of the aged individuals with overweight/obesity and metabolic syndrome.

## Supplementary Material

Physical activity shifts gut microbiota structure in aged subjects with overweight/obesity and metabolic syndrome

## Data sharing

According to the data regulations and ethical considerations, the datasets generated and analyzed during the study are not publicly available, because our participants only provided their consent to the original team of investigators to use their data, so this information might compromise their consent for the study. Nevertheless, collaboration for data analyses can be requested by sending a letter to the PREDIMED-Plus steering Committee (predimed_plus_scommittee@googlegroups.com). All members of the PREDIMED-Plus Steering Committee will be notified of the request for their consideration.
