# Supplementary material for: Physical activity shifts gut microbiota structure in aged subjects with overweight/obesity and metabolic syndrome
Source: Biol Sport. 2023 Dec 20;41(3):47–60. doi: 10.5114/biolsport.2024.133005 (PMC11167471; doi:10.5114/biolsport.2024.133005)
Supplement: Physical activity shifts gut microbiota structure in aged subjects with overweight/obesity and metabolic syndrome [file JBS-41-51833-s1.pdf]

SUPPLEMENTARY TABLE 1. Alpha diversity measurements in gut microbiota within each group T1, T2, and T3 after 1-year of intervention and, as well as, across the three groups.

	Faith_pd *p-value*	Observed features *p-value*	Pielou-Evenness *p-value*
**T1 (n = 99)**	0.981	0.722	0.319
**T2 (n = 99)**	0.701	0.780	0.574
**T3 (n = 99)**	0.675	0.720	0.055
**Across three groups**	0.438	0.758	0.122

SUPPLEMENTARY TABLE 2. List of the predicted pathways between three groups.

BioCyc Pathway ID	Pathway
**PWY-6562**	norspermidine biosynthesis
**BRANCHED-CHAIN-AA-SYN-PWY**	superpathway of branched chain amino acid biosynthesis
**ILEUSYN-PWY**	L-isoleucine biosynthesis I (from threonine)
**PWY-2942**	L-lysine biosynthesis III
**PWY-5101**	L-isoleucine biosynthesis II
**PWY-5103**	L-isoleucine biosynthesis III
**PWY-5104**	L-isoleucine biosynthesis IV
**PWY-6630**	superpathway of L-tyrosine biosynthesis
**VALSYN-PWY**	L-valine biosynthesis
**PWY-6628**	superpathway of L-phenylalanine biosynthesis
**PWY-5505**	L-glutamate and L-glutamine biosynthesis
**PWY0-1241**	ADP-L-*glycero*-β;-D-*manno*-heptose biosynthesis
**OANTIGEN-PWY**	*O*-antigen building blocks biosynthesis (*E. coli*)
**KDO-NAGLIPASYN-PWY**	superpathway of $(Kdo)_2$-lipid A biosynthesis
**PWY-5265**	peptidoglycan biosynthesis II (staphylococci)
**PWY-6891**	thiazole component of thiamine diphosphate biosynthesis II
**PWY-6895**	superpathway of thiamine diphosphate biosynthesis II
**PWY-7377**	cob(II)yrinate *a,c*-diamide biosynthesis I (early cobalt insertion)
**PYRIDNUCSAL-PWY**	NAD salvage pathway I (PNC VI cycle)
**LPSSYN-PWY**	superpathway of lipopolysaccharide biosynthesis
**P125-PWY**	superpathway of (*R, R*)-butanediol biosynthesis
**PWY-5910**	superpathway of geranylgeranyldiphosphate biosynthesis I (via mevalonate)
**PWY-5757**	fosfomycin biosynthesis
**PWY-922**	mevalonate pathway I (eukaryotes and bacteria)
**AEROBACTINSYN-PWY**	aerobactin biosynthesis
**ENTBACSYN-PWY**	enterobactin biosynthesis
**PWY-7431**	aromatic biogenic amine degradation (bacteria)
**CRNFORCAT-PWY**	creatinine degradation I
**ORNDEG-PWY**	superpathway of ornithine degradation
**PWY-5705**	allantoin degradation to glyoxylate III
**PWY-5499**	vitamin B_6 degradation

SUPPLEMENTARY TABLE 2. Continue.

**PWY0-1533**	methylphosphonate degradation I
**GALACTARDEG-PWY**	D-galactarate degradation I
**GLUCARDEG-PWY**	*D*-glucarate degradation I
**GOLPDLCAT-PWY**	superpathway of glycerol degradation to 1,3-propanediol
**AST-PWY**	L-arginine degradation II (AST pathway)
**ARGDEG-PWY**	superpathway of L-arginine, putrescine, and 4-aminobutanoate degradation
**ORNARGDEG-PWY**	superpathway of L-arginine and L-ornithine degradation
**BENZCOA-PWY**	anaerobic aromatic compound degradation (*Thauera aromatica*)
**GLUCARGALACTSUPER-PWY**	superpathway of D-glucarate and D-galactarate degradation
**PWY-6588**	pyruvate fermentation to acetone
**PWY-7111**	pyruvate fermentation to isobutanol (engineered)
**PWY-5100**	pyruvate fermentation to acetate and lactate II
**NONOXIPENT-PWY**	pentose phosphate pathway (non-oxidative branch) I
**PWY-7254**	TCA cycle VII (acetate-producers)
